# Individual quality and age but not environmental or social conditions modulate costs of reproduction in a capital breeder

**DOI:** 10.1002/ece3.3082

**Published:** 2017-06-15

**Authors:** Lucie Debeffe, Jocelyn Poissant, Philip D. McLoughlin

**Affiliations:** ^1^ Department of Biology University of Saskatchewan Saskatoon SK Canada; ^2^ College of Life and Environmental Sciences University of Exeter Penryn UK; ^3^ Present address: Centre for Ecological and Evolutionary Synthesis Department of Biosciences University of Oslo Oslo Norway

**Keywords:** *Equus ferus caballus*, heterogeneity, longevity, mammal, North Atlantic Oscillation, reproductive success, sociality, survival, ungulate

## Abstract

Costs associated with reproduction are widely known to play a role in the evolution of reproductive tactics with consequences to population and eco‐evolutionary dynamics. Evaluating these costs as they pertain to species in the wild remains an important goal of evolutionary ecology. Individual heterogeneity, including differences in individual quality (i.e., among‐individual differences in traits associated with survival and reproduction) or state, and variation in environmental and social conditions can modulate the costs of reproduction; however, few studies have considered effects of these factors simultaneously. Taking advantage of a detailed, long‐term dataset for a population of feral horses (Sable Island, Nova Scotia, Canada), we address the question of how intrinsic (quality, age), environmental (winter severity, location), and social conditions (group size, composition, sex ratio, density) influence the costs of reproduction on subsequent reproduction. Individual quality was measured using a multivariate analysis on a combination of four static and dynamic traits expected to depict heterogeneity in individual performance. Female quality and age interacted with reproductive status of the previous year to determine current reproductive effort, while no effect of social or environmental covariates was found. High‐quality females showed higher probabilities of giving birth and weaning their foal regardless of their reproductive status the previous year, while those of lower quality showed lower probabilities of producing foals in successive years. Middle‐aged (prime) females had the highest probability of giving birth when they had not reproduced the year before, but no such relationship with age was found among females that had reproduced the previous year, indicating that prime‐aged females bear higher costs of reproduction. We show that individual quality and age were key factors modulating the costs of reproduction in a capital breeder but that environmental or social conditions were not, highlighting the importance of considering multiple factors when studying costs of reproduction.

## 1 INTRODUCTION

Energy acquired by an individual can be allocated to survival, reproduction, or growth. Because energy is generally limited, individuals are expected to face trade‐offs when allocating resources between different fitness components (Stearns, 1992). In particular, because reproduction is energetically costly, reproductive events are expected to reduce future reproductive potential and/or survival (Reznick, 1985; Stearns, 1992; Williams, 1966). Costs of reproduction have been documented for many species (Clutton‐Brock, 1984; Gittleman & Thompson, 1988; Speakman, 2008), and are known to play a key role in the evolution of reproductive strategies (Roff, 2002) and population and eco‐evolutionary dynamics (Proaktor, Coulson, & Milner‐Gulland, 2008). Understanding the costs of reproduction in the wild and their role in the dynamics and evolution of species remains an important topic of evolutionary ecology.

Costs of reproduction are known to vary according to a species’ life history (Williams, 1966). Energy storage and how it is used is an important component of life‐history variation (Stearns, 1992), and income and capital breeders are the two extreme of a continuum of energy acquisition and use. Compared to income breeders that adjust their food intake concurrently with reproduction, capital breeders use energy stores for reproduction that have been accumulated at an earlier time (Jönsson, 1997; Stephens, Boyd, McNamara, & Houston, 2009). In long‐lived capital breeders, mothers usually favor their own growth and body maintenance over that of their offspring, resulting in negligible costs on an individual's own survival (Festa‐Bianchet & Jorgenson, 1998; Hamel, Cote, & Festa‐Bianchet, 2010). Nonetheless, reproduction can still reduce a mother's body condition (Gélin, Wilson, Coulson, & Festa‐Bianchet, 2015; Monteith et al., 2013; Simard, Huot, de Bellefeuille, & Cote, 2014; Testa & Adams, 1998) and future reproductive success (Festa‐Bianchet, Gaillard, & Jorgenson, 1998; Hamel, Gaillard et al., 2010; Moyes et al., 2011). Moreover, costs of reproduction can be influenced by a complex interplay among numerous intrinsic, environmental, and social factors (e.g., Hamel, Cote et al., 2010; Lescroel, Dugger, Ballard, & Ainley, 2009; Rauset, Low, & Persson, 2015; Robert, Paiva, Bolton, Jiguet, & Bried, 2012). However, to date few studies have considered multiple additive or interactive effects of these factors on the cost of reproduction.

Life‐history trade‐offs including costs of reproduction can be difficult to discern and explain in the presence of persistent among‐individual differences in performance—commonly referred to as “individual quality” (Cam, Link, Cooch, Monnat, & Danchin, 2002; Clutton‐Brock, 1984; Gélin et al., 2015; Weladji et al., 2008; Wilson & Nussey, 2010)—or in the context of short‐term or local environmental effects (Hamel, Yoccoz, & Gaillard, 2014). Variation in quality can have environmental (McNamara, 1998) or genetic (Nussey, Postma, Gienapp, & Visser, 2005) origins. For example, individuals who experience favorable environmental conditions early in life, known as “silver spoon” effects, can have reduced costs of reproduction throughout their lives (Vetter et al., 2016). Quality metrics have been criticized on the grounds of being ill‐defined and often not being comparable across studies (Bergeron, Baeta, Pelletier, Réale, & Garant, 2011; Moyes et al., 2009; Wilson & Nussey, 2010); however, when properly defined and interpreted they do provide insight into the biology of life‐history trade‐offs and costs of reproduction (Bridger, Bonner, & Briffa, 2015; Hamel, Cote, Gaillard, & Festa‐Bianchet, 2009; Hamel, Gaillard, Festa‐Bianchet, & Cote, 2009; Hassall, Sherratt, Watts, & Thompson, 2015; Tettamanti, Grignolio, Filli, Apollonio, & Bize, 2015). For example, in mountain goats (*Oreamnos americanus*), reproductive costs on future reproduction only became apparent after accounting for variation in a quality index based on covariation among longevity, success in the last breeding opportunity, adult mass, and social rank (Hamel, Cote et al., 2009).

Following Hamel, Cote et al. (2009), Hamel, Cote et al. (2010) and according to recommendations of Wilson and Nussey (2010) and Bergeron et al. (2011), individual quality may be defined as a covariation among life‐history traits at the individual scale, resulting in heterogeneity among individual performances within a population that are consistent throughout life. Although quality is often measured using a single life‐history trait (e.g., longevity, reproductive effort or success) or phenotypic trait, predicted to be linked to performance (e.g., body mass), two putative traits indexing quality might only be weakly correlated (Moyes et al., 2009; Wilson & Nussey, 2010) making preferable the use of several phenotypic traits (Bergeron et al., 2011; Wilson & Nussey, 2010), for instance by the means of multivariate analysis (see Hamel, Cote et al., 2009; Hamel, Gaillard et al., 2009 for examples).

In addition to variation in individual quality, which persists throughout an individual's lifetime, short‐term or local environmental conditions can also modulate costs of reproduction (Cam et al., 2002; Hamel, Cote et al., 2010; Robert et al., 2012). For instance, in a long‐lived seabird, the Monteiro's Storm‐Petrel (*Oceanodroma monteiroi*), costs of reproduction on adult survival were found to be mediated by oceanographic conditions surrounding the breeding colony (Robert et al., 2012). These authors also found that individual quality and oceanographic conditions interacted with each other, with higher costs among individuals of intermediate quality (Robert et al., 2012). If not accounted for, such variations might prevent the detection of reproductive costs in the study system.

While an increasingly large number of studies are reporting evidence for the effect of individual quality and environmental conditions on costs of reproduction (Cam et al., 2002; Clutton‐Brock et al., 1996; Hamel, Cote et al., 2009; Hamel, Cote et al., 2010; Lescroel et al., 2009; Robert et al., 2012; Weladji et al., 2008), little attention has been devoted to social effects such as group composition, sex ratio, or local density (Clutton‐Brock et al., 1996; Hamel, Cote et al., 2010; Nicolaus et al., 2012). In group‐living species, such social factors could be important in modulating costs of reproduction, but data on this is scarce. For example, there is evidence suggesting that the cost of raising offspring increases with density, as found in mountain goats, where a cost of reproduction on the probability of parturition was only apparent at high population density (Hamel, Cote et al., 2010). Other studies have shown that costs of reproduction can be influenced by local sex ratio. For example, in white‐faced capuchins (*Cebus capucinus*), female reproductive success was found to increase with the proportion of males in a group (Fedigan & Jack, 2011). In great tits (*Parus major*), local sex ratio affected the survival cost of reproduction with parents carrying small broods in male‐biased plots surviving better (Nicolaus et al., 2012). Together, these few reports highlight the need for considering local social environments in studies of the costs of reproduction.

**Figure 1:**
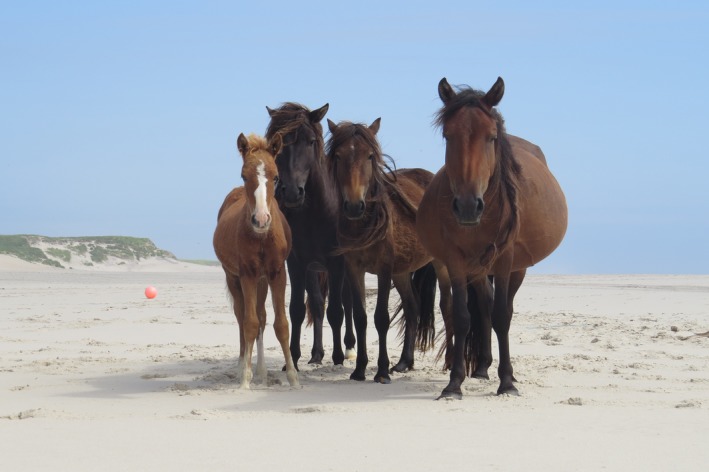
A band of Sable Island horses, Nova Scotia, Canada. © Photograph: L. Debeffe (2014)

Here, we investigated reproductive costs in females of a feral horse population (Sable Island, Nova Scotia, Canada; Figure 1). The population has been intensively monitored since 2007 (9 years of survey data, 2007–2015), and on average 35.3% of sexually mature females do not reproduce in a given year. Feral horses live in breeding groups (bands) that overlap in space and persist year round; their social system thus differs from most other polygynous ungulates and is more similar to that of some primates (Linklater, Cameron, Minot, & Stafford, 1999). The system presents an opportunity to address the question of how individual quality and environmental conditions, but also social conditions, might modulate the costs of reproduction in a capital breeder. In a large herbivore like the horse, we predict that reproductive expenditure may reduce future reproduction especially when resources are scarce (Jönsson, 1997; Stephens et al., 2009). Specifically, we tested if short‐term costs of reproduction (i.e., effects of reproductive effort the previous year on current probability of giving birth and weaning a foal) were modulated by intrinsic (age and quality) and extrinsic environmental (winter severity, location on the island) and social factors (band size, local density, band and local sex ratios [defined as the number of adult males divided by the number of adults in the band or the local area respectively]).

Based on previous research on other capital breeders, we predicted that costs of reproduction would be greater in poorer quality (Gomendio, Clutton‐Brock, Albon, Guinness, & Simpson, 1990; Hamel, Cote et al., 2009, 2010; Robert et al., 2012) and senescent (Clutton‐Brock, 1984; Hamel, Cote et al., 2010; McNamara & Houston, 1996) females (P1 and P2). We also expected environmental and social factors to modulate the cost of reproduction (Hamel, Cote et al., 2010; Nicolaus et al., 2012; Robert et al., 2012); more specifically, we predicted that the costs of reproduction would increase with winter severity (P3), local conspecific density (P4), and band size (P5). Under a high proportion of adult males, harassment toward females is known to be higher (Linklater et al., 1999); hence, we also predicted that females exposed to more adult males (in terms of local, relative sex ratio) would suffer greater reproductive costs (P6). Finally, because horse habitat quality decreases from west to east on Sable Island (forage quality and access to freshwater [Contasti, Tissier, Johnstone, & McLoughlin, 2012; Rozen‐Rechels et al., 2015]), we predicted that females in the east would bear greater reproductive costs (P7).

## 2 MATERIALS AND METHODS

### 2.1 Study area

Sable Island National Park Reserve (43°55′ N; 60°00′ W) is a vegetated sandbar located 275 km southeast of Halifax, Nova Scotia (Appendix S1). It is 49 km long and 1.3 km wide at its broadest point. The climate is temperate oceanic with warm summers and cool, wet and windy winters. Topography is characterized by sandy beaches, rolling heath meadows, and sand dunes that reach heights up to 30 m. The island's vegetation is dominated by marram grass (*Ammophila breviligulata*). The availability of freshwater as well as vegetation quality decrease from west to east (Contasti et al., 2012; Rozen‐Rechels et al., 2015). Horses in the west and central parts of the island have access to several permanent freshwater ponds, whereas horses in the east must excavate wells for access to freshwater. Introduced to Sable Island in the mid‐1700s, the horses are now the island's only terrestrial mammal (apart from a very limited human presence). While removals and supplementation occurred periodically over the last centuries, the population became legally protected from human interference in 1961 and has been unmanaged since then (Christie, 1995).

### 2.2 Data collection

#### 2.2.1 Population monitoring and life history

Population and life‐history data collection began with a partial census in the summer of 2007. From 2008 onward, systematic yearly ground censuses were performed during the mid–late breeding season (July to September). Daily walking censuses focused on one of seven sections, allowing complete coverage of the island in 1 week. The location of bands (i.e., breeding groups), bachelor groups, and lone individuals were recorded to within 5 m using a handheld Global Positioning System (GPS). At every encounter, multiple photographs were taken in addition to recording each horse's coloration, facial features, distinguishing marks, sex, age category (foal, yearling, 2–3 years old, or older), reproductive status, and group membership. Identifications were later confirmed in the laboratory using a photographic database. Sampling was performed under University of Saskatchewan Animal Care Protocol 20090032 and under guidance of the Canadian Council on Animal Care.

On average, each horse was observed 5 ± 2 times ($\overset{¯}{x}\pm SD$), with a maximum of 17 times per summer. Population counts (individuals known to be alive at September 1) from 2008 to 2015 were *n *=* *380, 437, 503, 448, 534, 559, 552, and 457, respectively. The entire dataset included 874 individuals including 600 foals born since 2007. From 2008 to 2015, more than 33,000 locations of horses were collected, and the likelihood of missing a horse if it was alive was very low (resighting probability across year: 0.994 for females and 0.992 for males). Hence, if a horse was not observed during an entire field season it was considered dead.

Using birth records and morphology we were able to accurately attribute birth year for individuals born after 2007 or first observed as yearlings and young adults (≤3 years old) in 2007 or 2008. For individuals first observed as adults (aged 4+ years) in 2007 and 2008 (53.1% of the females considered in this study), birth year was assumed to be 2002 and 2003, respectively. Note that winter 2002–2003 was characterized by a high mortality rate (with more than 280 carcasses found during the following summer), limiting our underestimation of the age of the individuals first seen as adult as we expected that many of the older adults may have died in 2002–2003. Our inability to age females first observed as adult accurately (*n *=* *60) was a weakness of the data; however, in order to limit any biases resulting from underestimating age at death, we ignored females that died within 2 years after first being observed as an adult (*n *=* *7). We retained for analyses only sexually mature females (3+ years old) with data available (as adult) for at least two successive years. Consequently, all females included in the analyses lived to at least 4 years of age. Further, to allow including longevity, age at last breeding, and success at last breeding event in a metric of quality, we only retained females that died over the study period (between 2007 and 2015) and were known to have reproduced at least once. By doing so we acknowledge that females dying old may have died at an underestimated age compared to females dying young; however, whereas reduced variability in longevity may limit our ability to detect an effect of quality it should not lead to false pattern. In total, our dataset contained 496 observations (births with foals surviving to end of summer, or not) from 113 females (4.39 ± 1.83 [($\overset{¯}{x}\;\pm \;SD$]).

Probability of giving birth to a foal (that successfully lived to September 1) and probability of weaning (survival to age 1) was assessed by observing females during our field season, which occurred during the mid–late breeding season (July–August). A female observed nursing a foal was characterized as having reproduced that year, and if the foal was still alive the following summer then the females was characterized as having weaned (recruited) the foal.

#### 2.2.2 Body condition

We assessed body condition of horses for inclusion into a metric of individual quality. Condition was assessed from photographs using the index of Carroll and Huntington (1988; see Debeffe et al., 2016 for details). The score reflects the amount of subcutaneous fat deposition on the hips, ribs, and spine, and ranges from zero (very poor condition) to five (obese). From an analysis using the dataset considered herein (not shown), we knew that body condition of adult females did not significantly change over a field season. Mean lifetime body condition of adult females was calculated as the average body condition obtained in different years (number of years with available body condition scores per female: ($\overset{¯}{x}\;\pm \;SD\;=\;4.38\;\pm \;1.84$, min = 1, max = 7, *n *=* *113).

#### 2.2.3 Individual quality

To determine if the approach of Hamel, Cote et al. (2009), Hamel, Gaillard et al. (2009) could be used to obtain a multivariate metric of quality in adult females, we tested if most of the covariation among four life‐history traits expected to be linked to lifetime fitness was captured by a single axis using a principal component analysis (PCA) with the R (R Development Core Team, 2010) ade4 package (Dray & Dufour, 2007). These traits were longevity, mean adult body condition, success at last breeding before death [three modalities: no reproduction code 0, reproduction but offspring died before the next summer code 1, and reproduction with offspring that survived code 2] and age at last reproduction). Longevity is often associated with performance in other life‐history traits (Nussey, Kruuk, Donald, Fowlie, & Clutton‐Brock, 2006) and is a commonly used trait to assess individual quality (Moyes et al., 2009; Espie, James, Oliphant, Warkentin, & Lieske, 2004; Hamel, Cote et al., 2009; Hamel, Gaillard et al., 2009; 2010; Weladji et al., 2006). Even if correlated, age at death and age at last reproduction differ for 28% of the females with a mean difference of 2.5 ± 1.05 years (maximum = 7 years). Specifically, we hypothesized that females who maintain high condition will also live longer, reproduce at a later age, and wean foals at an older age; whereas low condition females will either not live to an old age, or not attempt reproduction or successfully wean a foal when they do so. In contrast to other systems (e.g., Moyes et al., 2009), a clear threshold appeared when plotting the proportion of variation in traits accounted for by the successive principal components (PCs; see Appendix S2), with the first axis accounting for ~50% of the total variation. As predicted, covariation among the four traits was positive (Table 1). Following Hamel, Cote et al. (2009), Hamel, Gaillard et al. (2009), we therefore interpreted an individual's score on this PC as a description of its quality. High‐quality individuals were thus characterized by higher body condition, greater longevity, and having a higher probability of successfully breeding at their last attempt and of reproducing at an older age. As expected from an axis reflecting individual quality, PC1 was strongly correlated to a proxy of lifetime fitness (Wilson & Nussey, 2010), with higher quality females showing a higher number of reproductive events through the study period (linear regression: *n *=* *113, *R* ^2^ = 0.62, *F *=* *178.6, *df* = 111, *p *<* *.001).

**Table 1:** Scores, eigenvalue, and proportion of variance explained (%) for the first three axes (PC1, PC2, and PC3) of the principal component analysis (PCA) performed on four traits used to assess female quality in Sable Island horses (*n *=* *113)

Traits	PC1	PC2	PC3
Longevity	−0.95	−0.28	0.08
Age at last reproduction	−0.98	−0.09	−0.09
Success at last breeding attempt	−0.26	0.75	−0.61
Mean body condition score	−0.24	0.68	0.69
Eigenvalue	1.99	1.12	0.86
Variance explained (%)	49.65	27.93	21.49

#### 2.2.4 Environmental and social variables

We used the winter North Atlantic Oscillation (NAO; Hurrell & Van Loon, 1997) index as a proxy for winter severity. As described in Manning, Medill, and McLoughlin (2015), this was calculated as the geometric mean of daily NAO values from 1 January to 31 March, and developed into a continuous index using a *Z*‐score (standard normal random variable). The data were extracted from the National Oceanic and Atmospheric Administration's National Weather Service (available at: www.cpc.ncep.noaa.gov/products). On Sable Island, negative NAO index values generally reflect mild winter conditions characterized by high daily minimum and maximum temperatures, low number of days with frost, and low precipitation; while positive NAO index values reflect harsh winter conditions (see Appendix S3 for details). Because location on Sable Island is correlated with horse habitat quality (Contasti et al., 2012; Rozen‐Rechels et al., 2015), we also considered each individual's median longitude during summer census surveys as a measure of local environmental quality.

We considered band size and band sex ratio, as well as local (area) sex ratio and density as extrinsic social variables. Local density was calculated as the number of individuals (excluding foals) within 8,000 m of an individual's summer centroid location divided by the vegetated surface area (km^2^) within that buffer which is the area used by horses for foraging (number of horses per km^2^ of vegetated area; see Marjamäki, Contasti, Coulson, & McLoughlin, 2013 for more details). Local density was then calculated as a function of vegetated area (km^2^) preventing any bias induced by the presence of unequal surface of available area *versus* sea water inside the individual buffers. Local density was higher in the west compared to the east, and was highly correlated with habitat quality, with the best quality habitat supporting the highest densities (van Beest et al., 2014). We estimated band size as the mean number of individuals (excluding foals) present in an individual's band during the summer. We calculated local and band sex ratios as the number of males aged 3 years or more divided by the total number of individuals aged 3 or more within 8,000 m of an individual's summer centroid location (local sex ratio) or in the individual's band (band sex ratio), respectively (Manning et al., 2015). Sex ratio therefore ranges from one when only males are present to zero when only females are present.

### 2.3 Data analysis

We tested for reproductive costs and their determinants using univariate generalized linear mixed models implemented in the R package lme4 (Bates, Maechler, Bolker, & Walker, 2014). Specifically, we modeled a female's probability of giving birth (*n*_observations_ = 496, *n*_horses_ = 113) and of weaning a foal (i.e., foal surviving to the next summer for females having reproduced, *n*_observations_ = 321, *n*_horses_ = 113) using logistic regressions and then used a model selection approach to identify the best model. To test for costs of reproduction, reproductive status the previous year (produced a foal observed in July–August in the previous summer or not) was included as a fixed effect. To test for the influence of intrinsic and extrinsic variables on costs of reproduction, fixed effects also included female age (fitted as a continuous variable with a quadratic effect to allow for nonlinear relationships), female quality, median location in summer (standardized to a mean of 0 and *SD* of 1), band size, local abundance in summer (standardized to a mean of 0 and standard deviation of 1), local and band sex ratios, winter severity (winter NAO) before conception, during pregnancy and the first winter of the foal (only for models on the probability of weaning) as well as their two‐way interactions with reproductive status the previous year. More complex interactions were not considered to avoid overparameterization of our models. Horse identity was included as a random effect in all models to account for repeated measurements. Because local density and mean summer location were highly correlated (Pearson's product–moment correlation: correlation = 0.70, *n *=* *496, *t *=* *21.66, *df* = 494, *p *<* *.001), both factors were not included in the same model to avoid issues of colinearity. Because the relationship between female age and probability to give birth or wean a foal is not expected to be linear in feral horses (Garrott, Eagle, & Plotka, 1991), we added a second‐order polynomial term to allow for such nonlinearity. Note that band size and local density were not correlated (Pearson's product–moment correlation: correlation = –0.02, *n *=* *496, *t *= –0.45, *df* = 494, *p *=* *.65).

We fitted the global models described above as well as all simpler models in R using the AICcmodavg package (Mazerolle, 2015). The best models were then selected using the Akaike Information Criterion corrected for small sample size (AIC_c_), which reflects the best compromise between model precision and accuracy (Burnham & Anderson, 2002; Symonds & Moussalli, 2011). According to the rule of parsimony, we selected the simplest model within 2.0 AIC_c_ of the top model (Burnham & Anderson, 2002). We also calculated evidence ratio (ER) and AIC_c_ weights (AIC_c_Wts) as a measure of the likelihood that a given model was the best among the set of fitted models. Using the sum of the AIC_c_Wts (termed the predictor weight), we estimated the relative importance of each variable and interactions according to Symonds and Moussalli (2011). The predictor weight can be interpreted as being equivalent to the probability that the predictor is a component of the best model. For all selected logistic models we calculated the conditional and marginal coefficient of determination (*pseudo R* ^2^). The marginal coefficient of determination represents the variance explained by fixed factors and conditional coefficient of determination interprets as variance explained by both fixed and random factors (Nakagawa & Schielzeth, 2013).

## 3 RESULTS

### 3.1 Probability of having a live foal at the end of the summer

The probability of giving birth to a foal living to the end of summer (September 1) was best described by the model including reproductive status the previous year, female quality, female age, band sex ratio, band size, local density, and the two‐way interactions between female quality and reproductive status the previous year, and between female age and reproductive status the previous year (conditional *pseudo R* ^2^ = 0.19; marginal *pseudo R* ^2^ = 0.19; see Appendix S4a for details on model selection). The variables included in the selected model had the highest predictor weights (Table 2).

**Table 2:** Predictor weights calculated as the sum of the Akaike weights for each mixed‐effects logistic regression explaining variation in probability of giving birth to a foal surviving to late summer (*n*_horse_ = 113, *n*_observations_ = 496) or probability of weaning a foal (survival to year *t + *1; *n*_horse_ = 113, *n*_observations_ = 321) in which that variable appeared

Predictor	Analysis	
	Probability of giving birth	Probability of weaning
Repro *t* − 1	**1.00**	0.93
Female quality	**0.95**	**1.00**
Female age²	**0.96**	**1.00**
Location	0.00	0.01
Density	**1.00**	**0.98**
Band size	**1.00**	**0.94**
Local sex ratio	0.45	0.43
Band sex ratio	**0.97**	**0.80**
Winter severity *t* − 1	0.55	–
Winter severity	0.82	0.67
Winter severity *t + *1	–	**1.00**
Female quality: Repro *t* − 1	**0.73**	0.58
Female age²: Repro *t* − 1	**0.92**	0.19
Location: Repro *t* − 1	0.00	0.00
Density: Repro *t* − 1	0.43	0.28
Band size: Repro *t* − 1	0.37	0.27
Local sex ratio: Repro *t* − 1	0.12	0.21
Band sex ratio: Repro *t* − 1	0.38	0.37
Winter severity *t* − 1: Repro *t* − 1	0.14	–
Winter severity: Repro *t* − 1	0.22	0.17

Models included a combination of the following factors: female quality, female age, location on the island, local density, band size, band sex ratio, winter severity, and their interaction with reproductive status as fixed effects and horse identity as a random factor. Variables retained in the selected model are in bold.

**Figure 2:**
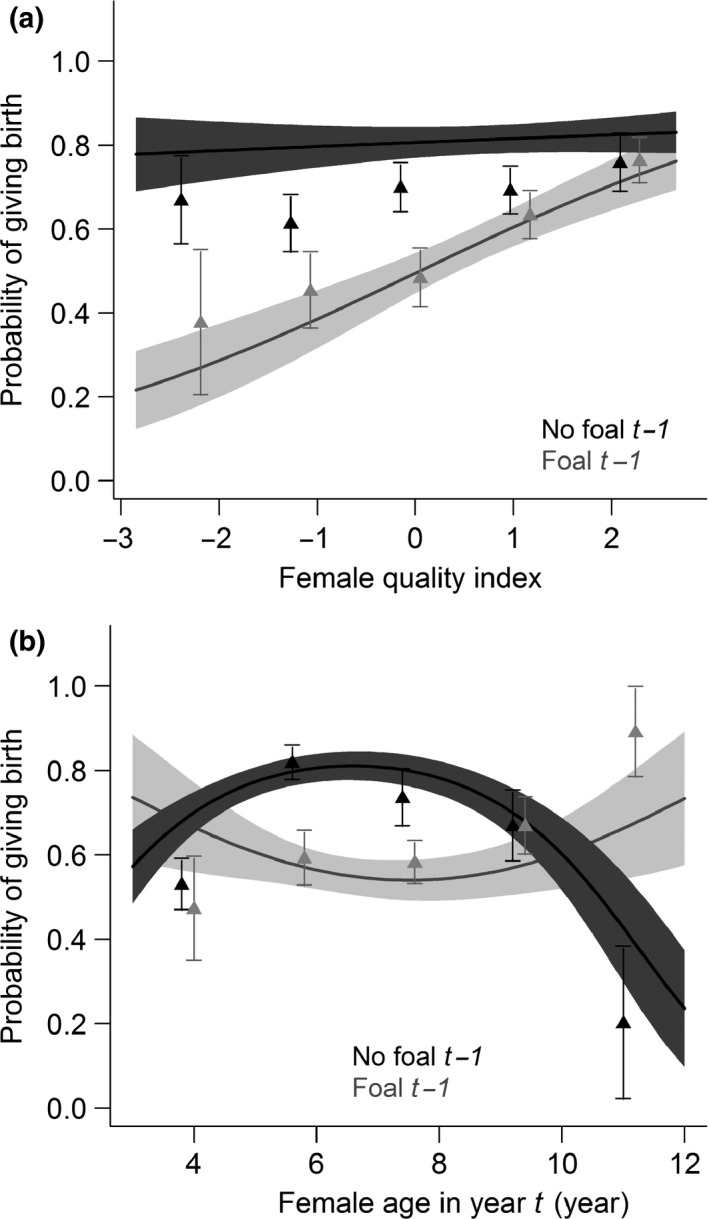
Relationships between a female's probability of giving birth to a foal surviving to late summer (September 1) in focal year *t* and (a) female quality, and (b) female age in year *t*, according to its reproductive status (“foal” or “no foal”) the previous year (*t* − 1). Here, *t* refers to the focal year (i.e., foal birth year). Models included local density, band size, band sex ratio, winter severity, and female quality or female age set to their mean values as fixed effects and horse identity as a random factor (*n*_horse_ = 113, *n*_observations_ = 496). The solid lines and the shaded areas depict relationships predicted by the selected models and their 95% confidence intervals, respectively

**Figure 3:**
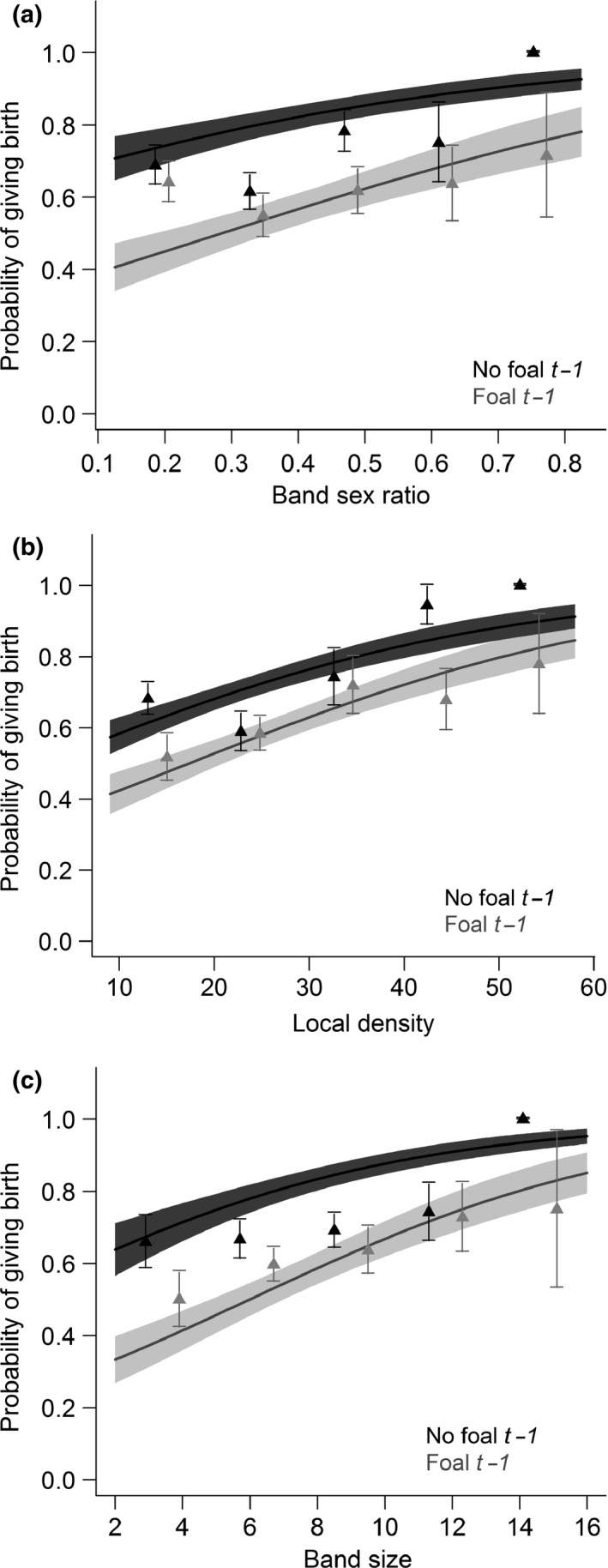
Relationships between a female's probability of giving birth to a foal surviving to late summer in focal year *t* and (a) band sex ratio, (b) local density, and (c) band size, according to its reproductive status (“foal” or “no foal”) the previous year (*t* − 1). Models included female quality, female age, winter severity and local density, band size, or band sex ratio set to their mean values as fixed effects and horse identity as a random factor (*n*_horse_ = 113, *n*_observations_ = 496). The solid lines and the shaded areas depict relationships predicted by the selected models and their 95% confidence intervals, respectively

Females that did not reproduce the previous year had a higher probability of giving birth in the focal year (Table 3a; predicted probability to give birth for females that did and did not reproduce the previous year, respectively: 0.57 ± 0.04 and 0.81 ± 0.04), highlighting some costs of reproduction on next reproduction. In addition, female quality and age interacted with the previous reproductive status, indicating that costs of reproduction were modulated by these variables (P1 and P2). Indeed, good‐quality females showed higher probabilities of giving birth than poor‐quality females regardless of their reproductive status the previous year, while poor‐quality females that reproduced the previous year showed a lower probability of giving birth compared to females that did not reproduce the previous year (P1; Table 3a; Figure 2a). Moreover, for nonreproductive females the previous year, the relationship between female age and the probability of giving birth was “bell‐shaped,” with younger and older females having a lower probability of giving birth while prime‐aged females (between 5 and 9 years old) had the highest (P2). We did not find this for females that reproduced the previous year (Table 3a; Figure 2b). Regardless of reproductive status the previous year, the probability of giving birth increased with band sex ratio bias toward males (Table 3a, Figure 3a), as local density increased (Table 3a, Figure 3b), and as band size increased (Table 3a, Figure 3c).

**Table 3:** Parameter estimates, associated standard deviation, *Z* value, and *p* value of the selected mixed‐effects logistic regressions for variation in (a) probability of giving birth to a foal surviving to late summer and (b) probability of weaning a foal (survival to year *t + *1)

Model variable	Estimate	*SD*	*Z*‐value	Pr(>|*z*|)	Odds ratio	OR‐LL	OR‐UL
(a) Probability of giving birth to a foal surviving to late summer							
Intercept	−1.05	0.55	−1.92	0.055	0.35	0.12	1.02
Quality	0.06	0.13	0.44	0.662	1.06	0.82	1.38
Repro *t* − 1 (Yes)	−0.84	0.25	−3.40	0.001	0.43	0.27	0.70
Female age, first‐order polynomial	−2.03	3.75	−0.54	0.589	0.13	0.00	206.00
Female age, second‐order polynomial	−10.80	3.22	−3.36	0.001	0.00	0.00	0.01
Density	0.36	0.09	3.87	<0.001	1.43	1.19	1.71
Band size	0.17	0.05	3.67	<0.001	1.18	1.08	1.29
Band sex ratio	2.31	0.82	2.82	0.005	10.10	2.02	50.20
Quality: Repro *t* − 1 (Yes)	0.39	0.20	1.90	0.057	1.47	0.99	2.20
Female age, first‐order polynomial: Repro *t* − 1 (Yes)	−0.79	6.58	−0.12	0.904	0.45	0.00	1.79E+05
Female age, second‐order polynomial: Repro *t* − 1 (Yes)	15.89	5.41	2.94	0.003	7.96E+06	197.00	3.22E+11
(b) Probability of weaning							
Intercept	3.91	1.07	3.65	<0.001	49.70	6.12	404.00
Quality	0.91	0.26	3.57	<0.001	2.49	1.51	4.10
Female age, first‐order polynomial	−18.60	5.59	−3.33	0.001	8.33E−09	1.47E−13	4.74E−04
Female age, second‐order polynomial	−5.16	3.18	−1.62	0.105	0.01	1.13E−05	2.92
Density	−0.50	0.14	−3.46	0.001	0.61	0.46	0.81
Band size	−0.22	0.08	−2.65	0.008	0.80	0.68	0.95
Band sex ratio	−2.58	1.41	−1.82	0.068	0.08	4.77E−03	1.21
Winter *t *+ 1	−0.95	0.24	−3.88	<0.001	0.39	0.24	0.63
Intercept	3.91	1.07	3.65	<0.001	49.70	6.12	404.00

Horse identity was included as a random factor in all models. *t* refers to the focal year (i.e., foal birth year). Odds ratio, odds ratio lower level (“OR‐LL”), and odds ratio upper level (“OR‐UL”) are also presented. See text for details about variables.

### 3.2 Probability of weaning

The probability to wean a foal (i.e., foal surviving to the next summer) for females having a live foal at the end of the summer was best described by the model including female quality, female age, winter severity after birth, local density, band size and band sex ratio (conditional *pseudo R* ^2^ = 0.55; marginal *pseudo R* ^2^ = 0.40; see Appendix S4b for details on model selection). The variables included in the selected model had the highest predictor weights (Table 2), except for the reproductive status the previous year that was not included in the selected model even with a high predictor weight.

**Figure 4:**
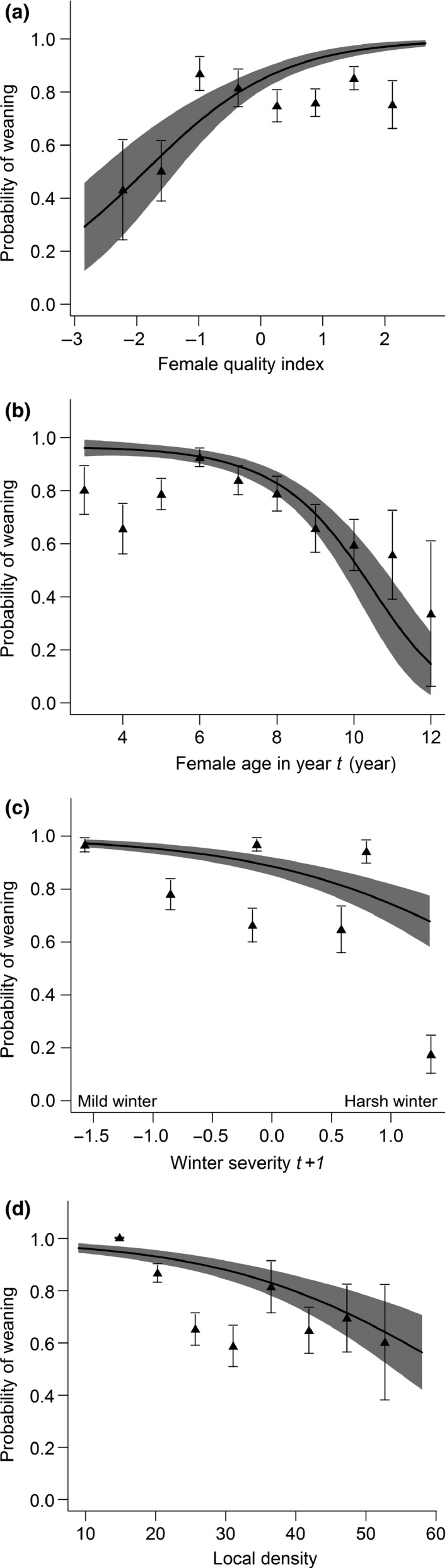
Relationships between a reproductive female's probability of weaning a foal (survival to year *t + *1) and (a) female quality, (b) female age in year *t*, (c) winter severity after birth, and (d) local density. The fixed effects not represented in the plots are set to its mean values and horse identity is included as a random factor (*n*_horse_ = 113, *n*_observations_ = 321). The solid lines and the shaded areas depict relationships predicted by the selected models and their 95% confidence intervals, respectively

No cost of previous reproduction on weaning success was detected for females having a live foal at the end of the summer. Good‐quality and young females showed a higher probability to wean a foal than poorer quality females (Table 3b, Figure 4a, b). Also, the probability to wean a foal increased as winter severity after birth decreased (Table 3b, Figure 4c), as local density decreased (Table 3b, Figure 4d), and as band size and sex ratio decreased (Table 3b).

## 4 DISCUSSION

Both the probabilities of giving birth to a foal that survived to the end of summer and weaning a foal overwinter to the next summer depended on intrinsic, environmental, and social factors. This is the first time these three categories of factors have been coupled in the same analysis to test for their effect on the costs of reproduction. Female quality and female age interacted with reproductive status in the previous year (P1, P2), highlighting their role in modulating the costs of reproduction on future reproduction, while no effect of social or environmental covariates was found (contradicting P3 to P7).

In Sable Island horses, the costs of reproduction were modulated by a female's individual quality. Good‐quality females showed higher probabilities of giving birth regardless of their reproductive status the previous year; whereas the probability to reproduce of poorer quality females was negatively affected by reproduction the previous year (Figure 2a). This suggests that good‐quality females were able to compensate for the costs of reproduction with no carryover effect on next reproduction; while in poorer quality females, reproduction entailed costs on their future reproduction. Restricting analyses on reproducing females, no such effects were detected on the probability of weaning, and previously reproductive females had the same probability to wean their foal. Higher probability of weaning was found for higher quality and younger females (Figure 4a, b). As with female quality, female age also modulated the cost of reproduction. Higher probabilities of giving birth (but not weaning an offspring) were found for prime‐aged females (P2) only if they had not reproduced the previous year, suggesting higher costs of reproduction for prime‐aged females; while particularly low probabilities were found for older females that did not previously reproduce (Figure 2b).

Female intrinsic variables as well as environmental and/or social factors influenced both the probability of giving birth and weaning an offspring. Indeed, the probability of giving birth increased with increasing local density and band size, and females in bands with a sex ratio biased toward males showed a higher probability of giving birth. The positive relationship found between winter severity and probability of giving birth suggests that contrary to expectation, the probability of giving birth was not constrained by winter severity before birth. Alternatively, higher overwinter foal mortality due to harsh winter conditions may enhance the probability of giving birth the following year by lowering abortion rates. However, the probability to wean the foal decreased as local density and band size increased. These effects of local density, band size, and band sex ratio highlighted the importance of considering social factors while studying the probability of giving birth and weaning an offspring.

Group size can have contrasting effects on reproductive success. For example, living in larger groups was shown to negatively affect reproductive success in black howler monkeys (*Alouatta pigra*; van Belle & Estrada, 2008), and to lower birth rates (but not offspring survival or interbirth interval) in Sichuan snub‐nosed monkeys (*Rhinopithecus roxellana*; Zhao, Li, & Watanabe, 2011) and long‐tailed macaques (*Macaca fascicularis*; van Noordwijk & van Schaik, 1999). Such negative consequences of group size have been attributed to the costs of sociality and in particular to increases in intragroup competition (van Noordwijk & van Schaik, 1999). However, living in large groups can also be advantageous. For instance, in a population of feral horses in the Llanos of Venezuela, Pacheco and Herrera (1997) noted a positive correlation between the number of females in a band and the number of foals per female born during the study. On Sable Island, Welsh (1975) highlighted a reduced number of births per female in small bands. As large bands form over long periods of time, we can expect that larger bands are more likely to be held by high‐quality, dominant and experienced stallions in feral horses. Indeed, bands increase in size during a stallion's early years of tenure (Welsh, 1975) and harem size is at its maximum for stallions between 6 and 9 years of age (Kaseda & Khalil, 1996). In Sable Island horses, the positive association between the probability to give birth and band size could be due to the presence of such dominant stallion, if these lead to lower harassment rates for females from outsider males, or the ability of experienced stallions to acquire resources or sheltering areas. Surprisingly, the reverse trend was found for the probability to wean the foal, highlighting that band size effect might be more complex and that factors affecting foal survival and female's probability to reproduce are different.

We found that the probability of giving birth increased as band sex ratio became more biased toward adult males. The same relationship was found in two primate species (Fedigan & Jack, 2011; Treves, 2001). The lower probability of giving birth in female‐biased bands might be due to increased female–female competition and aggression. Furthermore, the presence of more subadult and adult males in the group may lower the probability of take over from outsider males (Fedigan & Jack, 2011; Treves, 2001). Consequently, band stability seems to be a key factor influencing birth rate.

The probability of giving birth increased as local density increased. This finding is the opposite of what we expected in the presence of density‐dependent pressure (Gaillard, Festa‐Bianchet, Yoccoz, Loison, & Toigo, 2000). This counterintuitive result could be due to the benefits of being part of a large, presumably more stable band and held by an experienced stallion. Alternatively, such a pattern could be due to the process of domestication/feralization, where artificial selection for high reproductive performance in domestic species could cause females to trade their own survival for investment in reproduction under high density as found in Camargue horses (Grange, Duncan, & Gaillard, 2009). The probability to subsequently wean the foal, however, decreased as local density increased highlighting a negative density‐dependent foal survival.

Costs of reproduction have been found in many species (Gittleman & Thompson, 1988; Speakman, 2008), yet in some cases they have been difficult to detect (Toïgo et al., 2002; Weladji et al., 2008). As high‐quality females and/or females in suitable environmental and social conditions may be better at coping with the costs of reproduction than poor‐quality individuals and/or females living in less suitable conditions, heterogeneity in individual quality or environmental factors may be to blame for difficulties in detecting costs of reproduction. The probability of giving birth and of weaning increased with maternal quality in previously reproductive females, suggesting a greater cost of reproduction in poor‐quality females and highlighting the importance of female quality. In our specific case, without the addition of female quality, no costs of reproduction would have been detected (∆AIC_c_ to null model <2.0). Such links between individual quality and the costs of reproduction have been found in other studies (Hamel, Craine, & Towne, 2012; Hamel, Cote et al., 2009, 2010; Lescroel et al., 2009; Robert et al., 2012), and individual quality should, therefore, be taken into account while studying reproductive costs.

It is well established that the probability to reproduce and breeding success in females is not constant across ontogeny (McNamara & Houston, 1996). In several species, including horses, the relationship between the probability to reproduce or breeding success and female age has a typical bell shape, with prime‐aged females having the highest probabilities (Beauplet, Barbraud, Dabin, Kussener, & Guinet, 2006; Garrott et al., 1991; Rauset et al., 2015; Rughetti, Dematteis, Meneguz, & Festa‐Bianchet, 2015; Tettamanti et al., 2015). In Sable Island horses such a relationship was found, but only for females that did not reproduce the previous year. The difference between previously reproducing and nonreproducing females suggests that the cost of reproduction depends on a female's age, with prime‐aged females bearing higher costs of reproduction. Age‐dependent cost of reproduction was reported in several species, with higher costs of reproduction found in younger (i.e., first breeding) and/or older individuals (red deer, *Cervus elaphus* [Clutton‐Brock, 1984]; lesser snow geese, *Anas caerulescens caerulescens* [Viallefont, Cooke, & Lebreton, 1995]; greater flamingos, *Phoenicopterus ruber roseus* [Tavecchia, Pradel, Boy, Johnson, & Cezilly, 2001]; Soay sheep, *Ovis aries* [Tavecchia et al., 2005]; Weddell seals, *Leptonychotes weddellii* [Hadley, Rotella, & Garrott, 2007]; and wolverines [Rauset et al., 2015]). The probability of giving birth after a nonbreeding event is low in older animals (more than 10 years old; Figure 2a), and this decrease in fecundity and reproductive success in older individuals (i.e., senescence) is commonly observed in iteroparous species (Clutton‐Brock, 1984). However, contrary to expectations we did not find higher costs of reproduction in older (potentially senescent) females, and females that did actually reproduce after 10 years of age were more likely to reproduce again the following year compared to previously nonbreeding females. Similar results have been found in chamois (Morin, Rughetti, Rioux‐Paquette, & Festa‐Bianchet, 2016), where a strong tendency for some older females to reproduce successfully in consecutive years has been reported. Selective disappearance of the lower quality females through time could explain these patterns (Hamel, Cote et al., 2009).

We did not find evidence for effects of either environmental (winter severity, location) or social (local density, band size, local and band sex ratio) factors in modulating costs of reproduction. Costs of reproduction on both female survival (Festa‐Bianchet et al., 1998 in bighorn sheep; Tavecchia et al., 2005 in Soay sheep; Robert et al., 2012 in Monteiro's Storm‐Petrel) and future reproduction (Clutton‐Brock, 1984 in red deer; Rauset et al., 2015 in wolverines) were associated with environmental variation in other studies. For instance, reproductive costs were mediated by environmental harshness in a long‐lived seabird species, with unsuccessful breeders being more sensitive to oceanographic variation than more successful individuals (Robert et al., 2012). Moreover, reproductive costs on future reproduction were found to vary with population density in red deer (Clutton‐Brock, 1984) and mountain goats (Hamel, Cote et al., 2010). However, our results are similar to a study in female Weddell seals (*Leptonychotes weddellii*), where even in a highly variable environment, reproductive costs on future reproduction did not appear to vary substantially from year to year nor according to summer sea‐ice conditions (Hadley et al., 2007).

Our study highlights the importance of considering intrinsic, environmental, and social factors while investigating reproductive costs. We found that female quality and age were modulating costs of reproduction. Hence, in Sable Island horses, individual heterogeneity appears to be a key factor for costs of reproduction whereas social and environmental factors appear to play a lesser role. The presence of such effects may influence the probability of detecting costs of reproduction and should therefore be taken into account when possible. We call for additional research contrasting the effects of individual heterogeneity, environmental and social conditions on costs of reproduction.

## AUTHOR CONTRIBUTIONS

P. D. M. designed the study. L. D., J. P., and P. D. M. collected the data. L. D. analyzed the data and wrote the first draft of the manuscript. All authors contributed substantially to the final version.

## CONFLICT OF INTEREST

None declared.
