# Supplementary material for: Individual quality and age but not environmental or social conditions modulate costs of reproduction in a capital breeder
Source: Ecol Evol. 2017 Jun 15;7(15):5580–91. doi: 10.1002/ece3.3082 (PMC5552958; doi:10.1002/ece3.3082)
Supplement: Supplementary file 1 [file ECE3-7-5580-s001.docx]

**Supplementary Materials**

**Individual quality and age but not environmental or social conditions modulate costs of reproduction in a capital breeder**

Lucie Debeffe* ^a^, Jocelyn Poissant ^b^ and Philip D. McLoughlin ^a^

*^a^ Department of Biology, University of Saskatchewan, 112 Science Place, Saskatoon, SK S7N 5E2, Canada*

*^b^ College of Life and Environmental Sciences, University of Exeter, Cornwall Campus, Penryn, TR10 9FE, UK*

**Appendix S1. Map of the study site.**

Vegetated area

Non-vegetated area


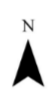

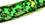


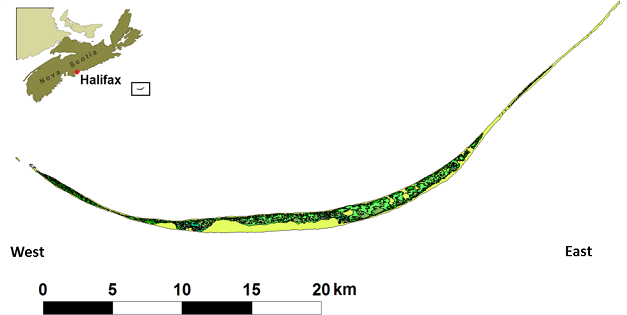


**Figure A1.** Map of Sable Island, Nova Scotia, Canada. The island is a crescent shaped sand bar 49 km long and 1.25 km at its widest.

**Appendix S2. Principal component analysis (PCA) to define female quality.**

**Figure A2.** Barplot of the eigenvalues of the PCA performed to defined female quality. Four traits were included in the analysis: Age at last breeding, longevity, success in the last breeding attempt (3 modalities: no reproduction code 0, reproduction but offspring died before the next summer code 1, reproduction with offspring that survive code 2), and mean body condition.

**Appendix S3. North Atlantic Oscillation index (NAO) and winter severity.**

Using climatic data from the weather station of Sable Island between 2006 and 2015 we tested for a relationship between NAO and winter severity on Sable Island (data are available at <http://climat.meteo.gc.ca/> and [www.cpc.ncep.noaa.gov/products](http://www.cpc.ncep.noaa.gov/products) for Sale Island and NAO data, respectively). The purpose of this analysis was to see to which extent the NAO, a global climatic index, reflects local climatic conditions. The NAO measures the strength of westerly winds blowing across the North Atlantic Ocean between 40° N and 60° N (Greatbatch 2000). Usually, high winter NAO index corresponds to milder winter conditions in the northeastern United States and southeastern Canada but harsher winter conditions in neighbouring Greenland, while low NAO correlates with harsher winter conditions (colder temperatures and heavy snowstorms) in the northeastern United States and southeastern Canada but milder winter conditions in Greenland. Given that Sable Island is located between these two geographic zones, it is not clear which air and water masses are mainly driving Sable Island winter conditions.

For each year between 2006 and 2015, we calculate the number of days with frost (i.e., with temperature lower than 0°C), mean minimum daily temperature, mean maximum daily temperature and total precipitations (mm) between January 1^st^ and March 31^st^. We performed a Principal Component Analysis (PCA) using the “ade4” R package (Dray & Dufour 2007) with the 10 years of data. A clear cut-off on the eigenvalues was apparent (Fig. A3), with the first axis (PC1) explaining 69% of the total variance (Table A1). Positive values on PC1 reflected harsh winter conditions characterized by low mean minimum and maximum temperatures, high number of days with frost, high precipitation and high mean NAO values (Table A1, Fig A4). In contrast, negative values on PC1 reflected mild winter conditions characterized by high mean minimum and maximum winter temperatures, low number of days with frost, low winter precipitation and were associated with low mean NAO values (Table A1, Fig A4). Positive NAO values over the winter were therefore associated with harsh winter condition on Sable Island while negative NAO values were associated with mild winter condition. For our analyses we used scores on PC1 as descriptors of winter severity.

**References**

Greatbatch, R. J. (2000). The North Atlantic Oscillation. *Stochastic Environmental Research and Risk Assessment*, **14**, 213-242.

**Table A1.** Scores, eigenvalues and variance explained (%) for the ﬁrst two axes (PC1 and PC2) of the Principal Component Analysis (PCA) performed on 2006-2015 winter NAO and local climatic data for Sable Island, Nova Scotia, Canada.

| ***Variable*** | ***PC1*** | ***PC2*** |
| --- | --- | --- |
| NAO | 0.81 | 0.53 |
| Total Precipitation | 0.79 | 0.55 |
| Frost days | 0.96 | -0.15 |
| Mean maximum temperature | -0.58 | 0.81 |
| Mean minimum temperature | -0.95 | 0.27 |
| Eigenvalue | 3.44 | 1.34 |
| Variance explained (%) | 68.89 | 26.78 |


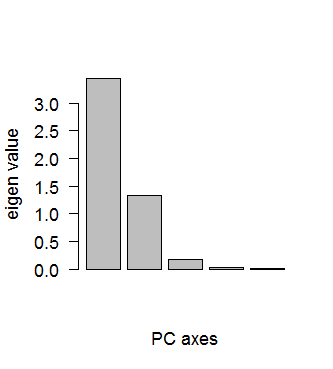


**Harsh winter**

**Mild winter**

**Figure A3.** Barplot of the eigenvalues for the five PC from the PCA performed on climatic data.

**Figure A4.** Circle of correlations between the 5 climatic variables used in the PCA on the first two axes (PC1 and PC2). Proximity of arrows reflects correlations between variables, with closer arrows showing positive correlations and opposing arrows reflecting negative correlations.


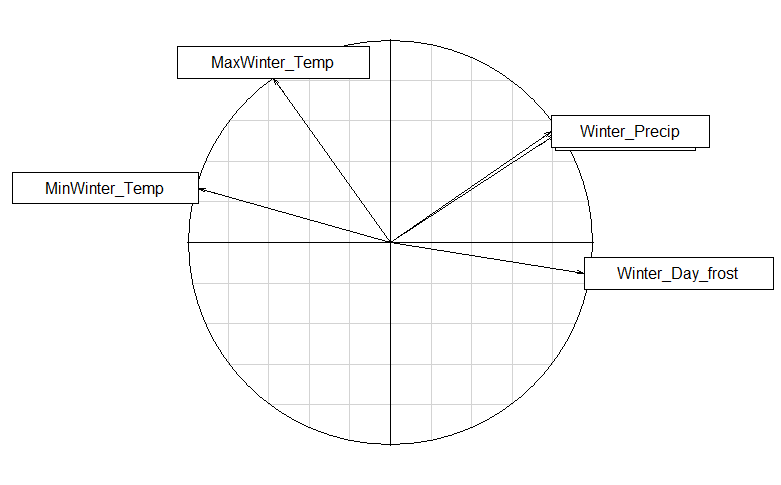


Winter_NAO

**Appendix S4. Model selection tables.**

**Table A2.** Model selection table for (a) probability of giving birth to a foal surviving to late summer in Sable Island adult females (*n* _horse_ = 113, *n* _observation_ = 496), and (b) probability of weaning a foal (*n* _horse_ = 113, *n* _observation_ = 321). The full model included fixed effects of female reproductive status the previous year (‘Repro *t–1*’), female quality index (‘Quality’), age (continuous with a quadratic effect, ‘Age²’), mean summer location along an east-west axis (‘Longitude) or local density (‘Density’), band size, winter severity before pregnancy (Winter *t–1*), before birth (‘Winter *t*’) and/or after birth (‘Winter *t+1’*) and their two-way interactions with female reproductive status the previous year. Horse identities were added as a random factor in all models. The loglikelihood (LogLik), corresponding AIC weight (AIC*c*Wt) and evidence ration (ER) are presented for all models with a ΔAIC*c* < 3. The candidate model sets (i.e., models with a ΔAIC*c* < 2) are in italic grey shade and the selected models are shown in bold.

| ***(a) Probability of giving birth*** | ***df*** | ***logLik*** | ***AICc*** | ***ΔAICc*** | ***AICc Wt*** | ***ER*** |
| --- | --- | --- | --- | --- | --- | --- |
| *Band Size + Density + Band SR + Winter t + Age²:Repro t–1 + Quality:Repro t–1* | *13* | *-287.96* | *602.68* | *0.00* | *0.02* | *1.00* |
| *Density + Band SR + Winter t + Band size:Repro t–1 + Age²:Repro t–1 + Quality:Repro t–1* | *14* | *-287.12* | *603.12* | *0.44* | *0.02* | *1.24* |
| *Band Size + Density + Winter t + Age²:Repro t–1 + Quality:Repro t–1 + Band SR:Repro t–1* | *14* | *-287.21* | *603.29* | *0.61* | *0.02* | *1.36* |
| *Band Size + Density + Band SR + Local SR + Winter t + Winter t–1 + Age²:Repro t–1 + Quality:Repro t–1* | *15* | *-286.24* | *603.48* | *0.80* | *0.01* | *1.49* |
| *Band Size + Density + Band SR + Winter t + Winter t–1 + Age²:Repro t–1 + Quality:Repro t–1* | *14* | *-287.32* | *603.51* | *0.83* | *0.01* | *1.51* |
| *Band Size + Band SR + Winter t + Density:Repro t–1 + Age²:Repro t–1 + Quality:Repro t–1* | *14* | *-287.34* | *603.56* | *0.88* | *0.01* | *1.55* |
| *Density + Band SR + Winter t + Winter t–1 + Band size:Repro t–1 + Age²:Repro t–1 + Quality:Repro t–1* | *15* | *-286.43* | *603.87* | *1.19* | *0.01* | *1.81* |
| *Band Size + Winter t + Density:Repro t–1 + Age²:Repro t–1 + Quality:Repro t–1 + Band SR:Repro t–1* | *15* | *-286.44* | *603.88* | *1.20* | *0.01* | *1.82* |
| *Band Size + Band SR + Local SR + Winter t + Winter t–1 + Density:Repro t–1 + Age²:Repro t–1 + Quality:Repro t–1* | *16* | *-285.43* | *603.99* | *1.31* | *0.01* | *1.93* |
| *Band Size + Density + Winter t + Winter t–1 + Age²:Repro t–1 + Quality:Repro t–1 + Band SR:Repro t–1* | *15* | *-286.50* | *604.00* | *1.31* | *0.01* | *1.93* |
| *Band Size + Band SR + Winter t + Winter t–1 + Density:Repro t–1 + Age²:Repro t–1 + Quality:Repro t–1* | *15* | *-286.51* | *604.02* | *1.33* | *0.01* | *1.95* |
| *Band SR + Winter t + Band size:Repro t–1 + Density:Repro t–1 + Age²:Repro t–1 + Quality:Repro t–1* | *15* | *-286.53* | *604.06* | *1.37* | *0.01* | *1.99* |
| *Density + Band SR + Local SR + Winter t + Winter t–1 + Band size:Repro t–1 + Age²:Repro t–1 + Quality:Repro t–1* | *16* | *-285.48* | *604.09* | *1.41* | *0.01* | *2.02* |
| *Band Size + Winter t + Winter t–1 + Density:Repro t–1 + Age²:Repro t–1 + Quality:Repro t–1 + Band SR:Repro t–1* | *16* | *-285.50* | *604.13* | *1.45* | *0.01* | *2.07* |
| *Band Size + Density + Local SR + Winter t + Winter t–1 + Age²:Repro t–1 + Quality:Repro t–1 + Band SR:Repro t–1* | *16* | *-285.53* | *604.20* | *1.52* | *0.01* | *2.14* |
| *Band Size + Density + Band SR + Local SR + Winter t + Age²:Repro t–1 + Quality:Repro t–1* | *14* | *-287.70* | *604.27* | *1.59* | *0.01* | *2.21* |
| *Band Size + Local SR + Winter t + Winter t–1 + Density:Repro t–1 + Age²:Repro t–1 + Quality:Repro t–1 + Band SR:Repro t–1* | *17* | *-284.54* | *604.36* | *1.68* | *0.01* | *2.32* |
| *Band SR + Winter t + Winter t–1 + Band size:Repro t–1 + Density:Repro t–1 + Age²:Repro t–1 + Quality:Repro t–1* | *16* | *-285.66* | *604.45* | *1.77* | *0.01* | *2.42* |
| ***Band Size + Density + Band SR + Age²:Repro t–1 + Quality:Repro t–1*** | ***12*** | ***-289.94*** | ***604.52*** | ***1.83*** | ***0.01*** | ***2.50*** |
| *Band SR + Local SR + Winter t + Winter t–1 + Band size:Repro t–1 + Density:Repro t–1 + Age²:Repro t–1 + Quality:Repro t–1* | *17* | *-284.69* | *604.66* | *1.98* | *0.01* | *2.69* |
| Band Size + Density + Band SR + Age²:Repro *t–1* + Quality:Repro *t–1* + Repro *t–1*:Winter | 14 | -287.93 | 604.73 | 2.05 | 0.01 | *2.79* |
| Density + Winter t + Band size:Repro *t–1* + Age²:Repro *t–1* + Quality:Repro *t–1* + Band SR:Repro *t–1* | 15 | -286.91 | 604.83 | 2.14 | 0.01 | *2.92* |
| Density + Band SR + Local SR + Winter t + Band size:Repro *t–1* + Age²:Repro *t–1* + Quality:Repro *t–1* | 15 | -286.92 | 604.84 | 2.16 | 0.01 | *2.95* |
| Band Size + Density + Local SR + Winter t + Age²:Repro *t–1* + Quality:Repro *t–1* + Band SR:Repro *t–1* | 15 | -287.01 | 605.02 | 2.34 | 0.01 | *3.21* |
| Band Size + Density + Quality + Band SR + Winter t + Age²:Repro *t–1* | 12 | -290.23 | 605.10 | 2.42 | 0.01 | *3.35* |
| Density + Band SR + Band size:Repro *t–1* + Age²:Repro *t–1* + Quality:Repro *t–1* + Repro *t–1*:Winter | 15 | -287.11 | 605.22 | 2.54 | 0.01 | *3.56* |
| Band Size + Band SR + Local SR + Winter t + Density:Repro *t–1* + Age²:Repro *t–1* + Quality:Repro *t–1* | 15 | -287.13 | 605.25 | 2.57 | 0.01 | *3.62* |
| Band Size + Density + Age²:Repro *t–1* + Quality:Repro *t–1* + Band SR:Repro *t–1* | 13 | -289.26 | 605.28 | 2.60 | 0.01 | *3.66* |
| Band Size + Density + Age²:Repro *t–1* + Quality:Repro *t–1* + Band SR:Repro *t–1* + Repro *t–1*:Winter | 15 | -287.19 | 605.37 | 2.69 | 0.01 | *3.84* |
| Density + Quality + Band SR + Winter t + Band size:Repro *t–1* + Age²:Repro *t–1* | 13 | -289.35 | 605.46 | 2.78 | 0.01 | *4.01* |
| Band Size + Quality + Band SR + Winter t + Density:Repro *t–1* + Age²:Repro *t–1* | 13 | -289.36 | 605.47 | 2.79 | 0.01 | *4.04* |
| Density + Winter t + Winter *t–1* + Band size:Repro *t–1* + Age²:Repro *t–1* + Quality:Repro *t–1* + Band SR:Repro *t–1* | 16 | -286.20 | 605.53 | 2.85 | 0.01 | *4.15* |
| Band Size + Density + Band SR + Winter t + Age²:Repro *t–1* + Quality:Repro *t–1* + Repro *t–1*:Winter *t–1* | 15 | -287.26 | 605.53 | 2.85 | 0.01 | *4.15* |
| Band Size + Density + Band SR + Winter t + Winter *t–1* + Age²:Repro *t–1* + Quality:Repro *t–1* + Local SR:Repro *t–1* | 16 | -286.20 | 605.53 | 2.85 | 0.01 | *4.16* |
| Density + Band SR + Band size:Repro *t–1* + Age²:Repro *t–1* + Quality:Repro *t–1* | 13 | -289.39 | 605.54 | 2.86 | 0.01 | *4.17* |
| Band Size + Density + Band SR + Local SR + Winter t + Age²:Repro *t–1* + Quality:Repro *t–1* + Repro *t–1*:Winter *t–1* | 16 | -286.20 | 605.54 | 2.86 | 0.01 | *4.17* |
| Band Size + Density + Band SR + Local SR + Winter *t–1* + Age²:Repro *t–1* + Quality:Repro *t–1* + Repro *t–1*:Winter | 16 | -286.21 | 605.56 | 2.88 | 0.01 | *4.21* |
| Winter t + Band size:Repro *t–1* + Density:Repro *t–1* + Age²:Repro *t–1* + Quality:Repro *t–1* + Band SR:Repro *t–1* | 16 | -286.22 | 605.57 | 2.89 | 0.01 | *4.24* |
| Band Size + Band SR + Density:Repro *t–1* + Age²:Repro *t–1* + Quality:Repro *t–1* + Repro *t–1*:Winter | 15 | -287.30 | 605.59 | 2.91 | 0.01 | *4.29* |
| Band Size + Density + Band SR + Winter *t–1* + Age²:Repro *t–1* + Quality:Repro *t–1* + Repro *t–1*:Winter | 15 | -287.31 | 605.62 | 2.94 | 0.01 | *4.34* |
| Band Size + Band SR + Density:Repro *t–1* + Age²:Repro *t–1* + Quality:Repro *t–1* | 13 | -289.44 | 605.64 | 2.95 | 0.01 | *4.38* |
| … | … |  |  |  |  |  |
| Constant | 2 | -321.99 | 648.01 | 49.11 | 1.35 E-12 | *4.61 E+10* |

| ***(b) Probability of weaning*** *(for reproducing females only)* | ***df*** | ***logLik*** | ***AICc*** | ***ΔAICc*** | ***AICc Wt*** | ***ER*** |
| --- | --- | --- | --- | --- | --- | --- |
| *Band size + Density + Age² + Winter t + Winter t+1 + Quality:Repro t–1 + Band SR:Repro t–1* | *13* | *-125.03* | *277.20* | *0.00* | *0.09* | *1.00* |
| *Band size + Density + Age² + Band SR + Winter t + Winter t+1 + Quality:Repro t–1* | *12* | *-126.27* | *277.50* | *0.30* | *0.08* | *1.16* |
| *Band size + Density + Age² + Winter t+1 + Quality:Repro t–1 + Band SR:Repro t–1* | *12* | *-126.60* | *278.20* | *0.96* | *0.06* | *1.62* |
| *Band size + Density + Age² + Band SR + Winter t+1 + Quality:Repro t–1* | *11* | *-127.85* | *278.50* | *1.30* | *0.05* | *1.92* |
| ***Band size + Density + Age² + Quality + Band SR + Winter t+1*** | ***9*** | ***-130.21*** | ***279.00*** | ***1.74*** | ***0.04*** | ***2.39*** |
| *Band size + Density + Age² + Local SR + Winter t + Winter t+1 + Quality:Repro t–1 + Band SR:Repro t–1* | *14* | *-124.86* | *279.10* | *1.84* | *0.04* | *2.51* |
| *Density + Age² + Band SR + Winter t + Winter t+1 + Band size:Repro t–1 + Quality:Repro t–1* | *13* | *-125.98* | *279.10* | *1.89* | *0.04* | *2.57* |
| *Band size + Density + Age² + Quality + Band SR + Winter t + Winter t+1* | *10* | *-129.18* | *279.10* | *1.82* | *0.04* | 2.48 |
| *Band size + Age² + Winter t + Winter t+1 + Density:Repro t–1 + Quality:Repro t–1 + Band SR:Repro t–1* | *14* | *-124.93* | *279.20* | *1.99* | *0.03* | 2.70 |
| *Band size + Density + Age² + Winter t + Winter t+1 + Quality:Repro t–1 + Band SR:Repro t–1 + Winter t:Repro t–1* | *14* | *-124.96* | *279.30* | *2.05* | *0.03* | *2.79* |
| Band size + Age² + Band SR + Winter t + Winter t+1 + Density:Repro *t–1* + Quality:Repro *t–1* | 13 | -126.08 | 279.30 | 2.10 | 0.03 | 2.86 |
| Density + Age² + Winter t + Winter t+1 + Band size:Repro *t–1* + Quality:Repro *t–1* + Band SR:Repro *t–1* | 14 | -125.03 | 279.40 | 2.18 | 0.03 | 2.97 |
| Band size + Density + Age² + Quality + Winter t + Winter t+1 + Band SR:Repro *t–1* | 12 | -127.21 | 279.40 | 2.18 | 0.03 | 2.97 |
| Band size + Density + Age² + Quality + Repro *t–1* + Band SR + Winter t + Winter t+1 | 11 | -128.27 | 279.40 | 2.14 | 0.03 | 2.92 |
| Band size + Density + Age² + Band SR + Local SR + Winter t + Winter t+1 + Quality:Repro *t–1* | 13 | -126.18 | 279.50 | 2.29 | 0.03 | 3.14 |
| Band size + Density + Age² + Band SR + Winter t + Winter t+1 + Quality:Repro *t–1* + Winter t:Repro *t–1* | 13 | -126.19 | 279.60 | 2.32 | 0.03 | 3.19 |
| Band size + Density + Age² + Winter t + Winter t+1 + Quality:Repro *t–1* + Band SR:Repro *t–1* + Local SR:Repro *t–1* | 15 | -124.05 | 279.70 | 2.43 | 0.03 | 3.37 |
| Band size + Density + Age² + Winter t + Winter t+1 + Quality:Repro *t–1* | 11 | -128.49 | 279.80 | 2.58 | 0.03 | 3.63 |
| Band size + Density + Age² + Band SR + Winter t + Winter t+1 + Quality:Repro *t–1* + Local SR:Repro *t–1* | 14 | -125.32 | 280.00 | 2.76 | 0.02 | 3.97 |
| Band size + Density + Age² + Local SR + Winter t+1 + Quality:Repro *t–1* + Band SR:Repro *t–1* | 13 | -126.42 | 280.00 | 2.77 | 0.02 | 3.99 |
| Band size + Density + Age² + Winter t+1 + Quality:Repro *t–1* + Band SR:Repro *t–1* + Local SR:Repro *t–1* | 14 | -125.38 | 280.10 | 2.89 | 0.02 | 4.24 |
| Band size + Age² + Winter t+1 + Density:Repro *t–1* + Quality:Repro *t–1* + Band SR:Repro *t–1* | 13 | -126.47 | 280.10 | 2.88 | 0.02 | 4.22 |
| Band size + Age² + Band SR + Winter t+1 + Density:Repro *t–1* + Quality:Repro *t–1* | 12 | -127.62 | 280.20 | 3.00 | 0.02 | 4.48 |
| Band size + Density + Age² + Quality + Winter t + Winter t+1 | 9 | -130.80 | 280.20 | 2.92 | 0.02 | 4.31 |
| Density + Age² + Winter t+1 + Band size:Repro *t–1* + Quality:Repro *t–1* + Band SR:Repro *t–1* | 13 | -126.58 | 280.30 | 3.10 | 0.02 | 4.71 |
| Band size + Density + Age² + Quality + Winter t+1 + Band SR:Repro *t–1* | 11 | -128.70 | 280.30 | 3.02 | 0.02 | 4.53 |
| Band size + Density + Age² + Quality + Repro *t–1* + Band SR + Winter t+1 | 10 | -129.78 | 280.30 | 3.02 | 0.02 | 4.53 |
| Band size + Density + Age² + Quality + Winter t+1 | 8 | -131.94 | 280.30 | 3.09 | 0.02 | 4.69 |
| Band size + Density + Quality + Winter t + Winter t+1 + Age²:Repro *t–1* + Band SR:Repro *t–1* | 14 | -125.50 | 280.40 | 3.13 | 0.02 | 4.78 |
| Density + Age² + Band SR + Winter t+1 + Band size:Repro *t–1* + Quality:Repro *t–1* | 12 | -127.68 | 280.40 | 3.13 | 0.02 | 4.78 |
| Band size + Density + Quality + Band SR + Winter t + Winter t+1 + Age²:Repro *t–1* | 13 | -126.59 | 280.40 | 3.12 | 0.02 | 4.76 |
| Band size + Density + Age² + Band SR + Winter t+1 + Quality:Repro *t–1* + Local SR:Repro *t–1* | 13 | -126.66 | 280.50 | 3.26 | 0.02 | 5.10 |
| Band size + Density + Age² + Band SR + Local SR + Winter t+1 + Quality:Repro *t–1* | 12 | -127.76 | 280.50 | 3.29 | 0.02 | 5.18 |
| … | … |  |  |  |  | 1.00 |
| Constant | 2 | -170.60 | 354.20 | 67.69 | 0.00 | 5.00E+14 |
